# Cosmetics and Detergents with Recycled CO_2_: A Cross-Country Study with a Modified by Risk Perception Values–Beliefs–Norms Model

**DOI:** 10.3390/bs13060518

**Published:** 2023-06-20

**Authors:** Irene Tilikidou, Antonia Delistavrou

**Affiliations:** Department of Organizations Management, Marketing and Tourism, International Hellenic University, 57400 Thessaloniki, Greece; irene.tilikidou@gmail.com

**Keywords:** values–beliefs–norms, climate change risk perception, consumer packaged goods, green chemicals, recycled CO_2_, moderation

## Abstract

This paper presents the examination of a values–beliefs–norms (VBN) model, modified by climate change risk perception, in France, Germany, and Spain, to investigate consumers’ intentions to purchase personal and house care products that are going to contain innovative ingredients made from recycled CO_2_. Electronic interviews were undertaken by a research agency on stratified (gender and age) samples in each country. Solely biospheric values indicated a statistically significant and positive causal relationship with risk perception. Risk perception provided the strongest of all impacts on awareness of consequences. Awareness of consequences affected the ascription of responsibility, and ascription of responsibility affected personal norms, which in turn generated consumption intentions. VBN was found powerful in explaining 58%, 60.2%, and 43.3% of the variance in intentions to buy CPGs with green chemical ingredients in French, German, and Spanish consumers, respectively. Moderation analysis indicated that the relationship between personal norms and consumption intentions is stronger in France and Germany than in Spain. Theoretical and practical implications are provided.

## 1. Introduction

Climate changes due to carbon emissions have been long ago considered to be one, if not the most, significant issue of environmental protection [1,2]. The planet’s global average surface air temperature has increased by about 1 °C (1.8° F) since 1900, with over half of the increase occurring since the mid-1970s, while the most recent decade (2010–2019) has been the warmest one since 1850, according to the available scientific measurements [3]. Carbon dioxide emissions have risen by about 50% since 1990 and increased more rapidly during the period 2000–2010, in comparison to the previous three decades, despite the fact that the 1990s was named the earth decade [4]. Among the other carbon gases, CO_2_ is the most polluting one, responsible for about 75% of the total emissions of greenhouse gases [5], and considered to be the leading cause of global warming [6], while bringing severe health issues for man [7].

Therefore, there is an urgent need to decrease carbon emissions towards the goal of neutrality [8]. Neutrality has been agreed to be both necessary and feasible by 2050, according to the documents of the UN Glasgow Financial Alliance for Net Zero [9]. 

### 1.1. Consumer Packaged Goods and Sustainability

Among other academic areas, the marketing discipline “needs and wants” to offer its own contribution to the overall goal of environmental protection. Moreover, it has been many times suggested that besides the necessary technological innovations, any pro-environmental project should include the economic and social dimensions of sustainable development [10,11]. Therefore, any low-carbon strategy and investment should be preceded by marketing research evidence regarding consumers’ intentions [8,12]. Environmental damages are considered to be partly rooted in consumption [13,14], whereas it has been previously suggested that they can be managed by changing the relevant behaviors to promote environmental quality [15]. Although there are discrepancies in the emissions calculation in the EU, the direct household emissions have been counted to be 18.8% of the total CO_2_ emissions in 2022 [16]. 

This study focuses on personal and household care products (cosmetics, shampoos, detergents, etc.) that count for a notable portion of the consumer packaged goods (CPGs) industry [17]. The importance of this every day-use category is self-evident, as it includes the final products that reach the consumer through all the production and distribution channels of industry and commerce, leaving a significant ecological footprint [18,19]. However, the relevant research on consumers’ attitudes and behavior remained rather scant [18,20]. There has been limited evidence indicating a niche but increasing market of consumers interested in ecologically friendlier cosmetics [21,22,23] and, to a lesser extent, detergents [24,25,26]. 

The attention of most researchers has been drawn to date by a distinct research stream concerning the so-called bio-cosmetics, namely organic or natural personal care products [27,28,29]. Nevertheless, it is to be taken into consideration that besides a rising tendency, natural and organic cosmetics still reach just a small portion of the European cosmetics market [30], while 95% are chemically produced products. There is a large research gap regarding synthetic cosmetics, even more so detergents. Nevertheless, they are produced in ways capable of reducing overall greenhouse gas emissions and thus qualify as sustainable products. The importance of interventions to reduce the pollution emitted by chemical industries is self-evident [11,31]. This study concerns chemical cosmetics and detergents, in which some of their conventional ingredients will be replaced by new, innovative oxo-products made from recycled CO_2_.

A pioneer reactor is being developed, which will capture CO_2_ from the atmosphere, recycle it with the help of sun and water, and use its components in the production of innovative, green oxo-products (https://suncochem.eu/ (accessed on 10 March 2023)). The recycled CO_2_ can be used in the production of three chemical sub-products, namely glycolic acid (useful in the production of personal care products), n-valeraldehyde (useful in the production of plastic and flavoring), and Limoxal^TM^ (useful in the production of fragrances, cosmetics, and household cleaning products). 

### 1.2. Significance and Aim

With regards to previous research efforts to explain consumers’ engagement in pro-environmental purchasing behavior, there has been extensive employment of a well-known model, namely the value–belief–norms (VBN) theory initiated by Stern and his colleagues [32,33]. In pro-environmental behavior research, VBN has been assumed to be driven by citizens’ values and beliefs that formulate moral obligations towards environmental protection for the benefit of society [34]. Therefore, it was judged suitable for this study as damages due to global warming affect societies in overall. Hence, consumers’ engagement in mitigating climate change is, by all means, a societally oriented subject. The question under consideration is whether specific consumer intentions to purchase new, sustainable cosmetics and detergents could be considered to be morally driven. 

The VBN theory suggests that pro-environmental behaviors are generated by personal norms that are activated in those persons, who would take responsibility to act towards mitigation of negative consequences of human behavior, believe in the necessity of a new environmental paradigm (NEP), while their beliefs are generated by their values concerning themselves, other people, and nature in general [32]. There have been numerous studies that followed the original model of VBN [35,36,37], while there have also been studies that modified or expanded VBN [6,38,39] in various topics. Further, although intentions are usually examined by the theory of planned behavior/TPB model [40], there have been modified models of VBN, in which purchasing intentions were examined, too [34]. 

There is a remarkable research gap in the employment of VBN so far regarding the examination of consumers’ intentions to get engaged in climate change mitigation, particularly regarding products containing recycled CO_2_. In this study, an expanded VBN model was employed to examine intentions perforce, as the new green sub-products are still in the research and development stage. There are many unanswered questions to date, such as whether the under-examination consumption intentions are morally driven or not. In case they are, which might be the most powerful values and beliefs in the chain of the VBN relationship, which are assumed to formulate norms and consequently intentions. 

To address this gap, in this modified VBN model, a replacement of NEP [41] is attempted by a variable nearer to climate change. The NEP is probably the most utilized measure of beliefs in pro-environmental research. Thus, it may not be expected that one more examination of NEP would significantly add anything to our relevant knowledge. Despite the valuable contribution of Dunlap and Van Liere [42] and Dunlap et al. [40], in the contemporary era, the results extracted by NEP have not been impressive, lying rather on the border of acceptable limits [34,43]. The examination of threats and risks due to global warming is expected to be more closely linked to innovative, green products capable of reducing CO_2_ emissions and contributing to climate change mitigation. Stern and his associates had made predictions that should be underlined as they regarded threats about environmentally related negative impacts to both human and nonhuman species as well as to the overall biosphere [44,45]. In the so far literature of risk perceptions, there has been one very robust construct, namely the index of climate change risk perception by Leiserowitz [46]. This construct particularly focuses on the examination of peoples’ understanding regarding current and future threats that are imposed to both nature and humankind by the constantly increasing temperature of the planet. Risk perception has been recently found able to predict actions aiming at mitigating climate change [47]. 

Therefore, the aim of this study was to test the power of a modified by risk perception VBN model to predict consumers’ intentions to buy cosmetics and detergents that are going to contain innovative, green ingredients made from recycled CO_2_, instead of the conventional chemicals. Further, it seemed promising to extend the examination in more than one country as differences in populations have been found able to provide significant variations in several pro-environmental variables [48,49]. Germany (DE), France (FR), and Spain (ES) were selected, as they are all among the largest European markets in sales of personal (DE: EUR 13.6 billion, FR: EUR 12.0 billion, ES: EUR 6.9 billion in 2021) and home care (projections for 2023 DE: EUR 6.3 billion, FR: EUR 5.7 billion, ES: EUR 3.6 billion) products [50,51]. 

## 2. Theoretical Framework and Hypotheses Setting

Stern and his associates [32,33] introduced the values–beliefs–norms (VBN) theory, which links the norm-activation theory (NAM) [52,53], the general values theory [45,54,55], and the new environmental paradigm (NEP) [42]. The VBN was designed as a theoretical framework, suitable to examine social movements and therefore ideal for the case of environmentalism [32]. The founders of VBN postulated that a coherent theory for examining any environmentally relevant behavior should be built on the values and beliefs that underlie its background [32,33]. The VBN research model [32] has been formulated on an alleged causal chain of five variables: specific, relatively stable human values (egoistic, altruistic, and biospheric) that affect awareness of consequences that in turn affect the ascription of responsibility, which, in conjunction with pro-environmental beliefs (NEP, [42]), would be able to activate personal norms leading to pro-environmental action [32,33]. There have been many studies to adopt and implement VBN in various topics [34,43,56], while there have also been numerous studies that modified VBN trying to understand better specific subjects in specific places and times [6,38]. The significance of the theoretical perspective of this study concerns the focus on the most important issue of environmental protection in our era, namely the mitigation of climate changes. In addition, it concerns the assumption that value-driven consumers’ intentions to contribute to the reduction of CO_2_ emissions are probably mostly enhanced by their perceptions about risks and threats due to global warming. The results are hopefully going to be of notable usefulness to a long list of shareholders, namely the EU and national policymakers, as well as chemical industries that are willing to produce the new, innovative oxo-products or utilize them in the production of cosmetics and/or detergents. 

**Figure 1:**
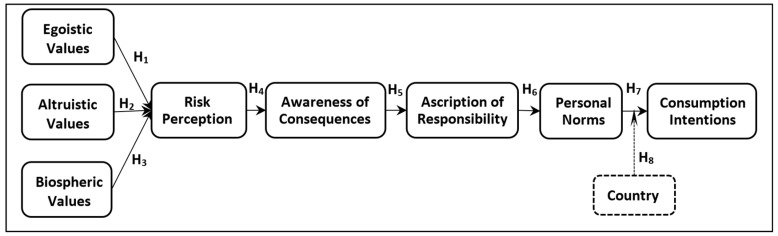
Theoretical Model.

Modifying VBN with risk perception, the theoretical framework of this study incorporates the three types of personal values (egoistic/EV, altruistic/AV, and biospheric values/BV), the risk perception (RP) [46], the more specific beliefs about negative conditions in the natural environment, namely awareness of consequences (AC) and the relevant humanity’s duty, namely ascription of responsibility (AR), leading to personal norms (PN) for pro-environmental action that are assumed able to predict consumption intentions (CI) towards cosmetics and detergents that are going to contain ingredients made from recycled CO_2_ (see Figure 1). Compared to the original Stern et al. [32] model, this framework’s main novelty is the replacement of the new environmental paradigm with climate change risk perception. Stern [33] suggested that each variable in the sequential chain of the VBN framework should be causally related to the next one, while it might also be found related to the following variables. Accordingly, the hypotheses setting in this study was formulated.

With regards to values, Stern et al. [57] worked on Schwartz’s [54] value typology and presumed a priori that many of Schwartz’s items reflected the egoistic, social-altruistic, and/or biospheric value orientations that had been identified by Stern et al. [44]. Later, these types have been found to influence NEP, customarily positively in the cases of altruistic and biospheric values and negatively in the case of egoistic values [34,35,58,59]. 

It is to be noted that there has not been a large enough amount of relevant, previous research with reference to the impact of environmentally related perceived risk on consumer behavior. Oreg and Katz-Gerro [60] incorporated some items of perceived threat (personal and general) that were found significantly and positively associated with willingness to sacrifice (pay more), whereas willingness to sacrifice was significantly and positively associated with environmental citizenship, recycling, and car non-use. Arbuckle et al. [61] examined the impact of perceived climate risks to agriculture and found that farmers’ beliefs in climate change as a “problem” impact significantly and positively on their willingness to adapt, while their attitudes regarding governmental support for mitigation (reduction of carbon emissions) appeared to be independent of perceived risks.

However, Leiserowitz [46] was the first to work on a reliable and valid index of climate change risk perception (CCRP). Leiserowitz’s [54] CCRP has been found to be related to pro-environmental behaviors [62,63,64,65,66]. The chosen construct consists of three sub-measures: i. global warming concern (holistic concern, seriousness of threat for nonhumans, and seriousness of current impacts around the world); ii. worldwide impacts of global warming on standards of living, water shortages and severe disease; and iii. local (at the place of the respondent’s living) impact of global warming on standards of living, water shortages and severe disease [67]. Stern argued that when a person’s values are under threat, beliefs and actions to mitigate these threats are more likely to be undertaken by this person [32]. Therefore, the first sub-measure of Leiserowitz’s [46] risk perception index, which particularly concerns the perception of threats and risks due to global warming, was considered to be ideal for this study. Climate changes threaten a person’s biospheric values, which may reflect his perception of risks due to global warming. 

The contribution of this study is expected to be both theoretical and practical. With regards to theory, it is expected to reveal that a variable closer to global warming (namely risk perception) would successfully replace the customary pro-environmental beliefs (NEP) to examine whether there is a sequence of values, beliefs, and norms that might be found able to describe and predict consumption intentions regarding CPGs containing oxo-products made from recycled CO_2_. With regards to practical contribution, the results of this study are going to hopefully provide useful information to several shareholders in the overall effort of sustainable development. Beneficiaries include EU policy makers, chemical industries that might be interested in producing oxo-products made from recycled CO_2_, and, finally, cosmetics and detergents industries that will find alternative, less-polluting chemicals for their production.

### 2.1. Values

Theoretically, it is assumed that if egoistic values are understood as a person’s care just for himself, this might be viewed as a self-enhancement orientation that has been viewed as the opposite of self-transcendence. Hence, it might be assumed that a person with strong egoistic values could not be sensitive to environmental deterioration [33]. An individual may believe that humankind naturally dominates the environment to provide resources to satisfy its own needs. Some people could also think that any threat due to climate change does not concern themselves but distant places and populations [46] and therefore be indifferent. Egoistic values have sometimes been found to negatively influence beliefs [35,68,69], while at other times, they have been found to be unrelated to beliefs and/or norms [34,70,71,72]. From the above points of view, it might be hypothesized that egoistic values rather restrict pro-environmental behavioral choices having a negative effect on any beliefs that generate pro-environmental personal norms. Therefore, the following hypothesis was set:

**H_1_.**  *Egoistic values are significantly and negatively related to risk perception.*

People, who are not only concerned about their own self-enhancement but value benefits and the welfare of other people, have been considered to be more likely to engage in pro-environmental behaviors [15,54,56,73]. Altruistic values primarily concern the effects of an individual’s behavior on other people’s benefits [33]. Altruistic values have been many times found to affect beliefs and norms positively [22,34,35,68,69,71,72,74,75]. Consequently, it is assumed that higher levels of altruism increase the strength of risk perception due to climate change, the negative effects of which harm all citizens in the community, nonetheless people in distant areas, too. Therefore, the following hypothesis was set:

**H_2_.**  *Altruistic values are significantly and positively related to risk perception.*

While altruistic values reflect a critical concern about other people’s risks or benefits, biospheric values reflect a closer concern with the status of the natural environment and other species beyond humans [15]. Such values emphasize the environment’s welfare and the necessity of preserving nature [76]. Essentially, they reflect an individual’s concerns about living in harmony with other species and nature as a whole. 

In this study, it is assumed that higher levels of biospheric values would primarily affect a person’s perception of threats and risks due to global warming. Indeed, biospheric values have been many times found to affect pro-environmental behavioral intentions more strongly [6,14,70] or exclusively [38]. Therefore, the following hypothesis was set.

**H_3_.**  *Biospheric values are significantly and positively related to risk perception.*

### 2.2. Beliefs

Although the worthiness of values in consumer behavior has been verified to be indisputable over time [77,78,79], the estimations of their impact on behavior have not been always found to be strong [80,81]. The so-called “value-action gap” had been previously attributed to the interference of other factors (e.g., beliefs, attitudes, or norms) in the direct relationship between values and behavior [56,82,83]. There has been a significant number of environmentally related studies, in which the interference of beliefs between values and norms and sequentially to behavior has been verified [42,59,72,84,85]. In particular, environmental awareness of environmentally unfriendly human activities, combined with consumer responsibility for global ecological consequences and the desire to mitigate them, have often been considered to lead to the sustainable consumption of several types [7]. 

Stern and his colleagues [57,86], based on Schwartz’s model [52,53], included in their model two types of beliefs, namely awareness of consequences (AC) and ascription of responsibility (AR). In the original VBN framework, an argument is made that AC is generated by NEP, and AR beliefs are generated by AC. In this study, it is postulated that AC should be generated by climate change risk perception. AC beliefs concern the acknowledgement that human activities are accountable for several environmental damages that threaten not just humankind but the biosphere in general [32]. This awareness represents a person’s understanding that environmental conditions will enhance or diminish the status of nature, humanity, and other species [33]. In this study, it is argued that the higher the climate change risk perception is, the higher the perceptions regarding consequences relevant to CO_2_ emissions due to CPGs’ production and consumption. Therefore, the following hypothesis was set:

**H_4_.**  *Climate change risk perception is significantly and positively related to awareness of consequences.*

The meaning of ascription of responsibility (AR) concerns the understanding of individuals’ responsibility for the threats and negative consequences due to their own activities [87]. Regarding the natural environment, AR refers to beliefs about the personal sense of responsibility that individuals should undertake to mitigate environmental damage [41]. It essentially means that people are aware of their own share of blame in terms of harmful consequences to both humans and the overall natural environment and that they are willing to take actions to adverse those consequences [56]. Thus, it is not just governments and other institutions responsible for the caused damages, but we ourselves should take responsibility for some negative consequences. In the particular subject of this study, it is examined whether respondents feel that they have contributed to the increase in CO_2_ emissions due to their own lifestyle. Therefore, the following hypothesis was set.

**H_5_.**  *Awareness of consequences is significantly and positively related to the ascription of responsibility.*

### 2.3. Personal Norms

Personal norms (PN) are customarily viewed as an individual’s feelings of moral obligations to perform or refrain from a specific action [43]. Schwartz [53] understood internalized or personal norms as different to social norms because the latter refer to expectations and obligations of groups while the first are anchored in the self alone. Zhang et al. [34] noted that personal norms are different from societal norms that have been introduced as an antecedent of intentions in the theory of reasoned action/TRA [88] and the theory of planned behavior/TPB [40]. Personal norms do not reflect the influence of important others on a person’s socially desirable obligations. Stern’s theorizing regards a value-expectancy perspective, arguing that when a person believes that one of his values is threatened and also believes that his actions are both responsible for the damage and capable of repairing it, that person feels compelled by his own value structure to act accordingly [32,89]. Nonetheless, Stern et al. [57] underlined that norms do not flow directly from the depths of a person’s psychology; an individual’s norms are generated by beliefs concerning specific issues of environmental protection, whereas they are activated in particular conditions. 

In subsequent research with VBN models, PN have been viewed as feelings of moral obligation generated by a person’s beliefs, mainly by the ascription of responsibility [43]. The relationship between responsibility and norms was found to be the strongest in some studies [34,72]. Nonetheless, in the overall picture of previous research, the results seem mixed in terms of the strongest evidence in the chain of relationships in VBN. In this study, it is examined whether ascription of responsibility impacts the respondents’ sense of obligation to buy CPGs containing green chemical ingredients in order to mitigate climate change. Therefore, the following hypothesis was set.

**H_6_.**  *Ascription of responsibility is significantly and positively related to personal norms.*

### 2.4. Intentions

Stern et al. [32] underlined that according to Schwartz’s [52,53] moral norm-activation theory of altruism, altruistic (including pro-environmental) behavior occurs in response to an individual’s moral norms. Stern [33] argued that the VBN theory should be viewed as the most powerful theory in predicting pro-environmental behavior (in comparison to other theories) because personal norms (PN) were found to be the stronger predictor for people’s predispositions to act pro-environmentally. In the course of time, there have been numerous applications of VBN providing evidence that PN is a significant predictor of pro-environmental behaviors [22,34,35,43,56,68,69,71,72,74,75]. 

With relevance to the specific subject of this research, it is to be noted that Stern [41] set an example concerning industrial products that might be “manufactured in more or less environmentally benign ways”. Further, it is to be noted that VBN had been deemed [32] to examine past or present behaviors (e.g., social movement and environmental activism), and indeed there have been numerous relevant applications [59,69,71]. Nevertheless, he suggested the examination of behavioral intentions, too. In fact, there have been many previous studies that applied VBN in the examination of pro-environmental behavioral intentions [34,36,38,58]. In this study, the variable “Consumption Intentions” was defined as the consumers’ intentions to prefer the new, innovative cosmetics and detergents that will contain ingredients made from recycled CO_2_. Therefore, the following hypothesis was set:

**H_7_.**  *Personal norms are significantly and positively related to consumption intentions.*

As mentioned in the Introduction, there are discrepancies in the market share of the personal and house care products among the largest European markets and significant variations in several pro-environmental consumer behavior variables [48,49]. In addition, according to Eurobarometer [90], the rates of EU citizens’ sense of responsibility also differ significantly (ES: 42%, FR: 46%, DE: 56%). Therefore, the following hypothesis was set:

**H_8_.**  *Country moderates the relationship between personal norms and consumption intentions.*

## 3. Materials and Methods

### 3.1. Sampling

The separate samples for the three countries were designed with the stratified sampling method according to the instructions of Zikmund [91] and Churchill and Iacobucci [92]. The strata were decided to be the gender and age distributions of each country’s population. A research agency was hired for the online data collection in all three countries, and the procedure was closely supervised by the authors of this paper. Besides the strata variables, data for other demographic variables, namely education, annual family income, and occupation, were also included in the questionnaire. Regarding education, it must be noted that population statistics are not available in a uniform format for each country, and thus education could not be initially included in the stratifying variables. Furthermore, the initial data collection provided over-representation of university and postgraduates in France and Spain. Therefore, additional gathering was asked of non-graduates in these two countries. The resulting sampling sizes and the response rates were: France 510 (25.02%), Germany 574 (68.23%), and Spain 454 (38.47%), while the sample demographic characteristics are presented in Table 1.

### 3.2. Variables Measurement

A structured questionnaire was developed in English and translated via the TRAP method (translation, review, adjudication, pretesting, and documentation) in the other three languages. It was decided to use scales with an even number of points (no midpoint). This type of measurement has been proposed as capable of forcing consumers to avoid neutral positions and to express a degree of agreement or disagreement [93,94]. In the cover letter, there was information that CPGs stand for green cosmetics and detergents (not food) that are going to contain chemical ingredients made from recycled CO_2_, instead of conventional chemicals. Additionally, there was a relevant reminder before the questions about beliefs and intentions.

The following variables were entered in the questionnaire: egoistic values (EV), altruistic values (AV), and biospheric values (BV) with four items each, all adopted from Steg et al. [56] measured on a 6-point importance scale. The first sub-measure of Leiserowitz’s [46] risk perception index, namely global warming concern with three items (RiskPer1), was measured on a 6-point rating scale. 

Awareness of consequences (AC) had five items, ascription of responsibility (AR) four items, and personal norms (PN) seven items. The phrasing of the items of the last three variables was based on Steg’s et al. [56] measures, while some were modified according to the topic under examination. In addition, the consumption intentions (CI) variable was added, including four items originally developed for this study. All items of the later four variables were measured on a 6-point Likert scale. The scale development procedure was based on instructions by Churchill [95,96], Robinson et al. [97], and Spector [98]. The initial item pool included 12 items gathered from one consumers’ and one experts’ focus groups, as well as from two elicitation techniques in students’ groups. The initial item pool was purified in the data of a preliminary students’ survey by the valorization of Cronbach’s alpha and item-to-total correlation techniques and resulted in four items. Expert colleagues were kindly asked to pre-test the overall questionnaire, in each one of the three languages. Their comments and suggestions were productively used to adapt the final questionnaires.

## 4. Results

The analyses were conducted by the utilization of SPSS v.17 and AMOS v.20 for covariance-based (CB) structural equation modeling (SEM) [99]. 

Before any statistical analyses were performed, the data were examined for missing values and outliers. No missing values were detected, and the Mahalanobis D^2^/df test [99] resulted in 12 outliers that were excluded. Thus, the sample was confined to 1526 cases (FR: 503, DE: 570, ES: 453). 

### 4.1. Demographics

Table 1 presents the demographics of each country. The gender and age distributions were compared to the relevant national statistics [100], and the t-test did not indicate any statistically significant differences. Nonetheless, it is observed that in the sample from France, the university graduates are somewhat more than those in the national distribution. 

### 4.2. Measurement Model

The initial measurement model analysis excluded four items (AC2, AC3, AC5, and AV2) due to factor loadings below the recommended threshold of 0.50 [99]. In addition, the construct of EV indicated statistically non-significant relationships with almost all other variables in Germany; thus, it was excluded from the final measurement model. The final measurement model was run in each country separately and obtained acceptable goodness-of-fit (GOF) values (Table 2), indicating that it fits the data very well. 

The unidimensionality of all constructs was tested by examining a) factor loadings, b) cross-loadings, and c) error covariances. All items’ factor loadings were above 0.60 (Table 2), while cross-loadings and error covariances were low.

The convergent and discriminant validity of the measurement model was examined in each country separately (Table 3). Convergent validity of all constructs was assessed as all (a) factor loadings were higher than 0.60, (b) average variances extracted (AVE) were higher than 0.50, and (c) construct reliabilities (CR) were all higher than 0.85, indicating exemplary reliability with the exception of AC (≥0.696) indicating good reliability [99,101]. Discriminant validity was tested with the heterotrait–monotrait (HTMT) ratios. All ratios were lower than the recommended threshold of 0.90 for constructs measuring similar concepts [102], indicating discriminant validity for all variables. Finally, nomological validity was assessed with statistically significant and positive correlations between all pairs of constructs (Table 3). 

As the research was conducted in different countries, measurement invariance was tested with configural and metric invariance assessment (Table 4). Multi-group configural invariance was assessed with the unconstrained model (all factor loadings were free to vary across groups). The GOF values indicated a close fit to the data (χ^2^ = 2096.732, df = 900, CFI = 0.964, TLI = 0.958, RMSEA = 0.030). Metric invariance was tested with the constrained model (factor loadings were constrained to be invariant across groups). The GOF values were close to those obtained in the unconstrained model (χ^2^ = 2163.758, df = 940, CFI = 0.963, TLI = 0.959, RMSEA = 0.029), and the Δχ^2^ test was not statistically significant (Table 4), indicating that all factor loadings are invariant across groups—countries. 

Common method variance was tested with the employment of Harman’s single factor test [103] by conducting exploratory factor analysis (EFA) in all items of all constructs, in each country. The common variance extracted for a single factor in the three countries was estimated to be 35.7% (FR), 37.7% (DE), and 34.9% (ES). All estimations were below the recommended 50% cut-off value, indicating that the data of this study do not suffer from common method bias. In addition, a second test for common method variance included the examination of the differences in multi-group GOFs [104] between the standard CFA and the random intercept CFA. The first criterion is the examination of the standard CFA fit according to the Hu’s and Bentler’s [105] cutoff values (RMSEA ≤ 0.05, SRMR ≤ 0.05, and CFI ≥ 0.95). The results of the standard CFA indicated a close fit to the data (RMSEA = 0.030, SRMR = 0.039, and CFI = 0.964). The second criterion is the examination of the differences between the standard CFA and the random intercept or the common unmeasured marker CFA with recommended cutoff differences (ΔRMSEA ≤ 0.015, ΔSRMR ≤ 0.030, and ΔCFI ≤ 0.010), indicating negligible common method effects [106,107]. The relevant test in our data indicated a ΔRMSEA of 0.001, ΔSRMR of 0.002, and a ΔCFI of 0.002 (random intercept CFA: RMSEA = 0.025, SRMR = 0.037, and CFI= 0.973). These results confirm that the cross-country data do not suffer from common method bias. 

As the validity of the measurement model was assessed in all countries, the examination of the structural model followed.

### 4.3. Descriptive Statistics

The means of the constructs (Table 2) indicated that respondents in each country hold a high level of both altruistic and biospheric values. They also hold a high level of the perceived risk about global warming and awareness of its consequences. However, they ascribe a moderate level of personal responsibility about emissions, global warming, and exhaustion of sources and a moderate level of personal norms to purchase CPGs with green chemicals. In sequence, they declared a moderate level of intentions to buy CPGs with ingredients made from recycled CO_2_. It is to be noted that the means of almost all constructs were slightly higher in the Spanish sample. 

### 4.4. Structural Model

The validity of the structural model was tested, and the GOF values indicated that it fits the data very well in each country (Table 5). The hypothesized consecutive relationships between BV, RiskPer1, AC, AR, PN, and CI were found to be statistically significant and positive. However, the hypothesized relationship between AV and RiskPer1 was found to be statistically non-significant in all countries (Table 5). These results led to the acceptance of H_3_, H_4_, H_5_, H_6_, and H_7_ and the rejection of H_2_. It is noted that EV was excluded from the final measurement model, and thus H_1_ is rejected. It is observed that the highest regression weights were found in the relationship between RiskPer1 and AC in all countries. 

Multi-group moderation analysis was conducted according to Hair’s et al. [99] and Byrne’s [108] guidelines to test whether there are statistically significant differences in the relationships between PN and CI across the three countries. Structural invariance was tested by comparing the unconstrained (relationships were freely estimated) and the constrained (paths were constrained to be invariant across countries) structural models. The GOF values obtained in the unconstrained structural model (Table 5) indicated a close fit to the data. Critical ratios out of the interval ±1.96 were found in the difference of the relevant coefficients between Spain and the other two countries (FR, DE). Then, the relevant path was modelled to be invariant across the three countries in the constrained model. The Δχ^2^ test was statistically significant (Table 5). Thus, H_8_ is accepted. The relationship between PN and CI is stronger in France and Germany than in Spain. 

The squared multiple correlations indicated that the modified VBN model is able to explain 58%, 60.2%, and 43.3% of the variance in intentions to buy CPGs with green chemical ingredients in French, German, and Spanish consumers, respectively. 

## 5. Discussion

The results of this study revealed that consumers’ intentions to purchase CPGs, which will contain ingredients manufactured with recycled CO_2_, were indeed found to be value driven, as they were to assumed to be. The theoretical framework that employed a modified VBN model was verified in the three European counties, in which it was applied, as impressive percentages of consumption intentions were found to be formulated by the sequence of values, beliefs, and norms. As expected, the country, when utilized as a moderator, revealed statistically significant differences among the three populations with regards to the percentage of variance explained in intentions. This finding is in line with the moderating effect of country that García-Salirrosas et al. [109] found among the Pacific Alliance countries.

It is to be noted that direct comparisons with previous research results are confined due to the differences in subject, place, and time but mainly due to the novelty of this study to replace NEP with risk perception. This novelty was found very meaningful in adding new perspectives, in our relevant knowledge so far, while the relevant results may very well be useful for several shareholders, mainly in Europe. 

Values are the first to be discussed, given the order of the links in the rationale of the VBN chain of relationships. There is a finding that regards values and differentiates this study from previous research results from the beginning. It concerns the unique role of biospheric values, as both egoistic and altruistic values were found insignificant in this application of VBN. At the preliminary stages of data processing, it was estimated that values, concerning an individual’s self, failed to correlate with the other variables, and thus egoistic values were excluded in the final measurement model. It should be noted that egoistic values have been excluded in some previous works, too, for example, those by Ünal et al. [6], Han et al. [70], and Hein [72]. More surprisingly, values regarding a person’s altruism to other people have not been found to determine his beliefs in this study, in none of the three countries. Although there have been a few recent studies in which altruistic values were excluded from the final VBN model [22], this finding is in contrast to the majority of previous research results [66,77,80] and specifically with regards to cosmetics [30,110]. Altruism has been a fundamental root of the VBN rationale and the level of altruism in all three countries was found to be rather high. It might be argued that values considering justice, equality, and mainly peace should be found to impact risk perception, especially since war causes the greater environmental disasters. In any case, the role of altruism is to be further evaluated, in future research efforts. At this point it is to be discussed that altruism concerns care for weak other people, who probably live in remote areas. It is to be noted that this study extended Steg’s et al. [14] prediction and Ünal’s et al. [6] findings that pro-environmental concerns, beliefs, attitudes, and behaviors are influenced mainly by biospheric values than by the other two types of values. The results of this study indicated that solely biospheric values are able to play a role in this application of VBN modified by risk perception. Overall, it is argued that values concerning the unity and harmony of humankind with nature are actually the only deeper roots of consumers’ intentions to contribute to the reduction of CO_2_ emissions through their purchasing choices. These observations might be linked, to an extent, with a rather distinct modification of VBN, that of value–identity–personal norms/VIP model [21,111]. VIP suggests that the extent to which individuals endorse biospheric values has an impact on their self-identity as an environmentally friendly person, which in turn motivates them to engage into various pro-environmental behaviors.

With regards to beliefs, it is underlined that the choice to replace NEP with risk perception should be considered successful and worthy of attention. It is reminded that NEP has recently indicated rather weak power to contribute to the formulation of norms and subsequently to behaviors [42,51], whereas, on the contrary, in this study, the power of risk perception was found to be impressive. Firstly, the level of risk perception was found to be high in all three countries. This is in contrast to Leiserowitz’s suggestion, back in 2006, that Americans’ perceptions concerned environmentally related dangers just with regards to nonhuman nature or people in geographically distant, underdeveloped places. Although France, Germany, and Spain are all considered to be affluent or at least developed countries, climate changes nowadays are understood by their populations as a threat to both human and nonhuman worlds. In this study, for the first time, to the best of our knowledge, it is revealed that climate change risks and threats are perceived to be out of the European citizens’ door. Secondly, the strength of the causal impact of risk perception on awareness of consequences was found to be the highest, among all the estimates of relationships in the chain that a VBN model requires, in all three countries. This is probably the main disclosure of this study and should be underlined. It reveals that risk perception being deeply rooted in people’s biospheric values is able to raise awareness of climate change consequences in consumers’ minds more strongly than any other variable in a VBN model has even been in previous research results. 

In the end, it should also be discussed that the weakest relationships in all three countries (mainly in Spain) were those regarding the impact of awareness of the consequences to the ascription of responsibility. This might be associated with the fact that the means of AR are lower than those of AC. These observations probably indicate that the step from understanding that human activities damage the environment to attributing real personal responsibility for restoration is difficult and needs to be further explored. This indication might be of further importance when considering that once this step it taken, the following one, which regards the impact of responsibility on personal norms, seems promising. 

## 6. Implications

### 6.1. Theoretical Implications

Theoretically, the main contribution of this study is the proposal to replace NEP with risk perception in VBN studies, especially those concerning consumers’ intentions to adapt their purchasing behavior with climate change mitigation. It should be added to our knowledge so far about risk perception that risks and threats of climate change are strongly perceived by citizens in the EU and cannot be viewed anymore as concerning people in some underdeveloped, remote countries. Therefore, it is implied that consumer research should focus more deeply on these perceptions of not just impending but present threats. In addition, the usual understanding of pro-environmental behavior, as mainly an altruistic-towards-others behavior, should be theoretically questioned. It is argued that not just any self-transcendent values (e.g., altruism) are able to maximize pro-environmental beliefs and not any self-enhancement values (e.g., egoism) are able to minimize them. It is suggested that academic research should focus on biospheric values as the major antecedent of risk perception. Further, not just any general pro-environmental beliefs are able to reveal the insights of the sequential chain that formulates intentions in VBN models. It is implied that the closer the assumed antecedents are to the core of the subject under examination, the better the resulting outcomes will be. It is evident that the key concept that adds importance to the examination of the sequence of values–beliefs–norms that formulates the pro-environmental consumer intentions is people’s perceptions about the risks of climate change that threaten nature, their own lives, and the lives of their children.

### 6.2. Practical Implications

With regards to practical implications, policymakers in European countries, as well as marketers in firms interested in producing and/or delivering green CPGs, would have much to gain from the results of this study. Firstly, they should all accept that the moderate estimation of intentions is plausible because cosmetics and detergents containing ingredients made from recycled CO_2_ have not yet been placed in the market. EU and national Green Deal strategies should be built on the main finding that risk and threats perceptions are—more than any other factor—able to enhance consumption intentions associated with mitigation of climate change. These intentions are built on a reverse sequence of norms, beliefs, and values. The most important links are those that are closer to the damages caused by carbon emissions. Special efforts in education and media should target the increase in values that concern the biosphere, as solely these were found to have an impact on risks due to global warming. Further, managers in industries interested in either producing the new oxo-products or in industries willing to use them (in the production of CPGs) are now provided with very useful knowledge regarding the formulation of consumption intentions. The information found can greatly facilitate the planning of their strategy once the new, innovative final products find their place in the market, as intentions are assumed to be the better antecedent of actual behavior. It is to be underlined that marketers had better take into consideration that consumers are not usually able to assess the ecological footprint of a product, especially the importance of certain production stages [112,113]. Therefore, creative messages should make evident, valid, and receivable of the fact that the new, innovative green products are indeed able to contribute to the mitigation of climate change. Effective smart communication tactics valorizing consumers’ biospheric values should emphasize the reduction of threats due to the reduction of CO_2_ in the atmosphere. These tactics, if attentively combined, will improve awareness of consequences, which eventually are hopefully going to increase the currently lower levels of responsibility and norms that formulate intentions and consequently actual behavior in the near future.

## 7. Limitations and Further Research Suggestions

As in any self-reported subject concerning public and natural welfare, it is acknowledged that the results of this study may suffer from a social desirability (SD) effect [114]. Although bias metrics have been satisfactory, it is suggested that a separate, distinct scale of SD would serve better in the elimination of the relevant effect in future studies. Further, a limitation of this study is the perforce examination of intentions and not actual behavioral preferences. Once the new, innovative cosmetics and detergents (or any other CPGs containing ingredients made from recycled CO_2_) appear in the market, further research efforts are required to provide actual estimations of consumer behavior. Another limitation is that just the country of residence was used as a moderator in this study, while in future studies, other mediators or moderators might be found helpful in explaining larger parts of the variance in the dependent variables under examination. Moreover, modified by climate change risk perception VBN models should be applied in other European and non-European countries. Furthermore, it is to be noted that there have been previous suggestions [60] implying that both values and attitudes should be encompassed in the examination of pro-environmental behaviors. In recent research, there have been some studies that incorporated combined models of VBN and TPB [38,39,68] in a non-stop effort to understand better both moral and rational antecedents of pro-environmental intentions and behaviors. Further, the distinct modification of VBN, namely value–identity–personal norms/VIP model [13,111] might provide fruitful insights if combined with risk perception.

The CO_2_ recycling is evidently of particular importance as it does not concern any controversial measure of green development, such as the carcinogenic incineration of waste or the degradation and commercialization of forests for installing giant wind farms and turbines. Therefore, it is argued that there is much to be expected from future research concerning the productive use of recycled CO_2_.

## 8. Conclusions

This study aimed to fill a part of the research gap so far by employing VBN for the first time to examine consumption intentions towards CPGs, specifically designed to reduce already-emitted CO_2_, extending VBN by replacing NEP with climate change risk perception, which has also been underutilized in the examination of consumer pro-environmental intentions, to date. Consumption intentions (CI) regarding cosmetics and detergents that are going to contain new, green ingredients made from recycled CO_2_ were examined in France, Germany, and Spain by the employment of a VBN model modified by climate change risk perception. Biospheric values were found able to affect positively and significantly risk perception, while altruistic values (AV) were not. Egoistic values (EV) were excluded from the model due to the lack of nomological validity. It was found that the stronger causal relationship in all countries is the one that concerns the effect of risk perception (RiskPer1) on awareness of consequences (AC). AC was found to affect significantly and positively the ascription of responsibility (AR), while AR affected personal norms (PN) in all three countries. It seems that in Germany, all relationships are stronger (impacts of RiskPer1 on AC, AC on AR, AR on PN, and PN on CI) except for the effect of BV on RiskPer1, which is stronger in France. Moderation analysis indicated that the relationship between personal norms (PN) and consumption intentions (CI) is stronger in France and Germany than it is in Spain. VBN was found powerful in explaining 58%, 60.2%, and 43.3% of the variance in intentions to buy CPGs with green chemical ingredients in French, German, and Spanish consumers, respectively. It seems that the modified VBN model of this study provided stronger explanatory power in Germany and France than it did in Spain. 

Therefore, this study, in which consumption intentions were for the first time examined with regards to CPGs containing oxo-products made from recycled CO_2_, contributed to our knowledge so far in pro-environmental consumer behavior due to the replacement of NEP with risk perception in a modified VBN model. It was revealed that biospheric values is the only antecedent of risk perception while risk perception impacting impressively to awareness of consequences and in sequence to ascription of responsibility and personal norms is the key factor able to enhance consumption intentions. 

There have been many claims that the COVID-19 pandemic would have driven trends towards responsible behavior and enhance green consumption [115,116,117]. Nonetheless, we should mention that the COVID-19 pandemic and the ongoing energy crisis caused an apparent delay in the implementation of the agreements on climate as in any other projects on environmental protection [118]. Moreover, although threats concerning dangers due to global warming are ranked high in people’s concerns, the working classes are justifiably worried primarily about the threats regarding income restrictions or unemployment. This is why the European Green Deal must emphasize the concept of a fair transition, in case we may still consider that such a concept is possible in a world where the pursuit of profit is the absolute priority. “If green transition is not going to be fair, it is just not going to happen at all”, Frans Timmermans, vice president of European Commission, underlined [119]. 

## Figures and Tables

**Table 1:** Demographic characteristics.

	France		Germany		Spain	
	n	%	n	%	n	%
Total	503	100	570	100	453	100
***Gender***						
Men	242	48.2	286	50.2	226	49.9
Women	261	51.8	284	49.8	226	49.9
Other					1	0.2
***Age***						
18–24 years old	62	12.3	68	11.9	61	13.4
25–34 years old	97	19.3	117	20.5	67	14.8
35–44 years old	109	21.7	107	18.8	94	20.8
45–54 years old	103	20.5	102	17.5	95	21.0
55–64 years old	63	12.5	98	17.2	85	18.8
65 years or older	68	13.5	80	14.0	51	11.3
No answer	1	0.2				
***Education***						
Primary school	11	2.2	10	1.8	25	55
Secondary school	192	38.2	53	9.3	148	32.7
Vocational training	72	14.3	334	58.6	112	24.7
University	127	25.2	116	20.4	105	23.2
Masters	82	16.3	43	7.5	51	11.3
Ph.D.	10	2.0	9	1.6	12	2.6
No answer	9	1.8	5	0.9		
***Annual Income***						
up to EUR 5.000	31	6.2	23	4.0	22	4.9
between EUR 5.001–15.000	54	10.7	66	11.6	73	16.1
between EUR 15.001–25.000	96	19.1	79	13.9	131	28.9
between EUR 25.001–35.000	110	21.9	100	17.5	109	24.1
between EUR 35.001–45.000	86	17.1	88	15.4	55	12.1
between EUR 45.001–55.001	55	10.9	67	11.8	22	4.9
EUR 55.001 and more	40	8.0	108	18.9	25	5.5
No answer	31	6.2	39	6.8	16	3.5
***Occupation***						
Professional/Entrepreneur/Farmer	75	14.9	38	6.7	47	10.4
Private employee	102	20.3	252	44.2	125	27.6
Public employee	74	14.7	35	6.1	57	12.6
Unemployed	52	10.3	18	3.2	43	9.5
Houseperson	21	4.2	37	6.5	35	7.7
Retired	88	17.5	110	19.3	58	12.8
Student	35	7.0	31	5.4	49	10.8
Other	45	8.9	37	6.5	32	7.1
No answer	11	2.2	12	2.1	7	1.5

**Table 2:** Measurement Model: GOFs, Factor Loadings, and Means.

	GOF Values						
	χ^2^	df	Sig.	χ^2^/df	TLI	CFI	RMSEA
**France**	661.941	300	*p* < 0.001	2.206	0.961	0.966	0.049
**Germany**	858.017	300	*p* < 0.001	2.860	0.951	0.958	0.057
**Spain**	576.801	300	*p* < 0.001	1.923	0.965	0.970	0.045
					**France**	**Germany**	**Spain**
					**Factor Loadings**		
***Altruistic Values*** *(**AV**)* (range 3–18)				Mean	14.085	14.368	15.252
AV1	Social justice: correcting injustice, care for the weak				0.766 ***	0.754 ***	0.706 ***
AV3	Equality: equal opportunity for all				0.856 ***	0.863 ***	0.850 ***
AV4	A world at peace: free of war and conflict				0.815 ***	0.832 ***	0.876 ***
***Biospheric Values*** *(**BV**)* (range 4–24)				Mean	18.600	19.417	20.260
Bio1	Protecting the environment: preserving nature				0.892 ***	0.899 ***	0.891 ***
Bio2	Preventing pollution				0.909 ***	0.862 ***	0.884 ***
Bio3	Respecting the earth: live in harmony with other species				0.850 ***	0.888 ***	0.875 ***
Bio4	Unity with nature: fitting into nature				0.818 ***	0.870 ***	0.854 ***
***Risk Perception 1*** *(**RiskPer1**)* (range 3–18)				Mean	13.135	13.281	14.340
RP1	How concerned are you about global warming?				0.823 ***	0.890 ***	0.802 ***
RP2	How serious of a threat do you believe global warming is to nonhuman nature?				0.914 ***	0.905 ***	0.860 ***
RP3	How serious are the current impacts of global warming around the world?				0.838 ***	0.924 ***	0.843 ***
***Awareness of Consequences*** *(**AC**)* (range 2–12)				Mean	9.141	8.951	9.717
AC1	Global warming has consequences for society				0.861 ***	0.830 ***	0.864 ***
AC4	The exhaustion of energy sources is a problem				0.660 ***	0.623 ***	0.619 ***
***Ascription of Responsibility*** *(**AR**)* (range 4–24)				Mean	16.089	16.039	16.349
AR1	I am jointly responsible for CO_2_ emissions				0.833 ***	0.812 ***	0.775 ***
AR2	I feel jointly responsible for the exhaustion of energy sources				0.892 ***	0.878 ***	0.898 ***
AR3	I feel jointly responsible for global warming				0.926 ***	0.894 ***	0.896 ***
AR4	Not only the government and industry are responsible for high levels of CO_2_ emissions, but me too				0.679 ***	0.747 ***	0.747 ***
***Personal Norms*** *(**PN**)* (range 7–42)				Mean	25.525	25.772	27.528
PN1	I feel personally obliged to buy CPGs containing green chemical ingredients				0.813 ***	0.799 ***	0.788 ***
PN2	Regardless of what others do, I feel morally obliged to buy CPGs containing green chemical ingredients				0.783 ***	0.823 ***	0.783 ***
PN3	I feel guilty when I do not buy CPGs containing green chemical ingredients				0.715 ***	0.801 ***	0.777 ***
PN4	I feel morally obliged to use ecological products instead of regular products				0.847 ***	0.844 ***	0.822 ***
PN5	When I buy a new CPG, I feel a moral obligation to prefer one that contains green chemical ingredients				0.882 ***	0.878 ***	0.868 ***
PN6	People like me should do everything they can to buy CPGs containing green chemical ingredients				0.830 ***	0.854 ***	0.820 ***
PN7	I would be a better person if I consumed CPGs containing green chemical ingredients				0.828 ***	0.719 ***	0.744 ***
***Consumption Intentions*** *(**CI**)* (range 4–24)				Mean	15.843	16.160	17.404
CI1	I will buy CPGs containing green chemical ingredients if they are of similar quality to the regular products				0.769 ***	0.757 ***	0.701 ***
CI2	I will buy CPGs containing green chemical ingredients if they are of similar price to the regular products				0.818 ***	0.660 ***	0.732 ***
CI3	I am seriously thinking to buy CPGs containing environmentally friendlier ingredients as soon as I run out of the products I am currently using				0.846 ***	0.883 ***	0.858 ***
CI4	I will definitely switch to a brand of a CPG that contains green chemical ingredients				0.813 ***	0.853 ***	0.816 ***

*** *p* < 0.001.

**Table 3:** Measurement Model: Reliability and Validity.

	Cronbach Alpha	CR	AVE	Correlations HTMT Ratios					
				*AV*	*BV*	*RiskPer1*	*AC*	*AR*	*PN*
**FRANCE**									
***Altruistic Values*** *(**AV**)*	0.852	0.854	0.661						
***Biospheric Values*** *(**BV**)*	0.924	0.924	0.753	0.774 *** 0.774					
***Risk Perception*** *(**RiskPer1**)*	0.890	0.894	0.738	0.442 *** 0.443	0.668 *** 0.669				
***Awareness of Consequences*** *(**AC**)*	0.724	0.738	0.588	0.535 *** 0.539	0.651 *** 0.657	0.786 *** 0.793			
***Ascription of Responsibility*** *(**AR**)*	0.897	0.903	0.702	0.155 ** 0.155	0.426 *** 0.426	0.573 *** 0.575	0.568 *** 0.574		
***Personal Norms*** *(**PN**)*	0.936	0.933	0.665	0.140 ** 0.020	0.346 *** 0.343	0.448 *** 0.444	0.429 *** 0.428	0.678 *** 0.672	
***Consumption Intentions*** *(**CI**)*	0.898	0.885	0.659	0.271 *** 0.266	0.438 *** 0.429	0.505 *** 0.495	0.469 *** 0.463	0.531 *** 0.520	0.760 *** 0.736
**GERMANY**									
***Altruistic Values*** *(**AV**)*	0.855	0.858	0.669						
***Biospheric Values*** *(**BV**)*	0.932	0.932	0.774	0.852 *** 0.853					
***Risk Perception*** *(**RiskPer1**)*	0.931	0.933	0.822	0.528 *** 0.529	0.666 *** 0.666				
***Awareness of Consequences*** *(**AC**)*	0.679	0.696	0.539	0.686 *** 0.694	0.722 *** 0.729	0.818 *** 0.828			
***Ascription of Responsibility*** *(**AR**)*	0.900	0.901	0.697	0.372 *** 0.373	0.482 *** 0.482	0.719 *** 0.720	0.725 *** 0.734		
***Personal Norms*** *(**PN**)*	0.937	0.934	0.670	0.242 *** 0.240	0.382 *** 0.378	0.605 *** 0.599	0.581 *** 0.581	0.768 *** 0.761	
***Consumption Intentions*** *(**CI**)*	0.887	0.870	0.629	0.357 *** 0.346	0.432 *** 0.419	0.573 *** 0.556	0.581 *** 0.570	0.583 *** 0.566	0.774 *** 0.743
**SPAIN**									
***Altruistic Values*** *(**AV**)*	0.849	0.854	0.663						
***Biospheric Values*** *(**BV**)*	0.930	0.930	0.768	0.841 *** 0.843					
***Risk Perception*** *(**RiskPer1**)*	0.873	0.874	0.698	0.530 *** 0.531	0.679 *** 0.679				
***Awareness of Consequences*** *(**AC**)*	0.697	0.716	0.565	0.630 *** 0.640	0.672 *** 0.681	0.829 *** 0.841			
***Ascription of Responsibility*** *(**AR**)*	0.897	0.899	0.692	0.264 *** 0.265	0.334 *** 0.334	0.537 *** 0.538	0.520 *** 0.527		
***Personal Norms*** *(**PN**)*	0.929	0.926	0.642	0.225 *** 0.224	0.349 *** 0.345	0.521 *** 0.516	0.485 *** 0.486	0.723 *** 0.717	
***Consumption Intentions*** *(**CI**)*	0.878	0.860	0.607	0.344 *** 0.334	0.413 *** 0.400	0.498 *** 0.482	0.490 *** 0.481	0.522 *** 0.506	0.653 *** 0.625

CR: Construct reliability, AVE: Average variance extracted, HTMT: Heterotrait–monotrait, *** *p* < 0.001, ** *p* < 0.05.

**Table 4:** Measurement Invariance.

	χ^2^	Δχ^2^	df	Δdf	Δχ^2^/Δdf	Sig.
Configural Invariance (Unconstrained model)	2096.732		900			
Metric Invariance (Constrained model)	2163.758	67.026	940	40	1.675	*p* > 0.05

**Table 5:** Structural Models, Structural Invariance, and Hypotheses testing.

	Structural Models				Structural Invariance	
	France	Germany	Spain		Unconstrained Model	Constrained Model
	**GOFs**				**GOFs**	
χ^2^	774.285	962.210	666.377		2402.846	2411.340
sig.	*p* < 0.001	*p* < 0.001	*p* < 0.001		*p* < 0.001	*p* < 0.001
df	314	314	314		942	944
χ^2^/df	2.466	3.064	2.122		2.551	2.554
TLI	0.952	0.946	0.957		0.951	0.959
CFI	0.957	0.951	0.961		0.956	0.956
RMSEA	0.054	0.060	0.050		0.032	0.032
	**Structural relationships** *(β)*				**Δχ^2^ test**	
***EV → RiskPer1***	EV excluded in the measurement model			H_1_: Not supported	Δχ^2^	8.494
***AV*** → ***RiskPer1***	−0.109 n.s.	−0.099 n.s.	−0.078 n.s.	H_2_: Not supported	Δdf	2
***BV*** → ***RiskPer1***	0.818 ***	0.762 ***	0.763 ***	H_3_: Supported	Δχ^2^/Δdf	4.247
***RiskPer1*** → ***AC***	0.843 ***	0.951 ***	0.882 ***	H_4_: Supported		(*p* < 0.05)
***AC*** → ***AR***	0.631 ***	0.762 ***	0.583 ***	H_5_: Supported		
***AR*** → ***PN***	0.684 ***	0.774 ***	0.736 ***	H_6_: Supported		
***PN*** → ***CI***	0.762 ***	0.776 ***	0.658 ***	H_7_: Supported		
*Critical Ratios*	*DE: 0.181*	***ES: −2.751***	***FR: −2.528***	H_8_: Supported		
	**Squared Multiple Correlations** (**R^2^**)					
	0.580	0.602	0.433			

*β*: standardized regression weights, *** *p* < 0.001, n.s.: non-significant.

## Data Availability

Data available on request due to restrictions. The data presented in this study are available on request from the corresponding author. The data are not publicly available due to a privacy agreement introduced with the informed consent.
